# Implications of lncRNAs in *Helicobacter pylori*-associated gastrointestinal cancers: underlying mechanisms and future perspectives

**DOI:** 10.3389/fcimb.2024.1392129

**Published:** 2024-07-05

**Authors:** Lei Zhang, Fei Yu, Yue Zhang, Peifeng Li

**Affiliations:** Institute for Translational Medicine, The Affiliated Hospital of Qingdao University, Qingdao University, Qingdao, China

**Keywords:** *H. pylori*, lncRNAs, gastrointestinal cancer, mechanisms, clinical perspective

## Abstract

*Helicobacter pylori* (*H. pylori*) is a harmful bacterium that is difficult to conveniently diagnose and effectively eradicate. Chronic *H. pylori* infection increases the risk of gastrointestinal diseases, even cancers. Despite the known findings, more underlying mechanisms are to be deeply explored to facilitate the development of novel prevention and treatment strategies of *H. pylori* infection. Long noncoding RNAs (lncRNAs) are RNAs with more than 200 nucleotides. They may be implicated in cell proliferation, inflammation and many other signaling pathways of gastrointestinal cancer progression. The dynamic expression of lncRNAs indicates their potential to be diagnostic or prognostic biomarkers. In this paper, we comprehensively summarize the processes of *H. pylori* infection and the treatment methods, review the known findings of lncRNA classification and functional mechanisms, elucidate the roles of lncRNAs in *H. pylori*-related gastrointestinal cancer, and discuss the clinical perspectives of lncRNAs.

## 1 Introduction

*Helicobacter pylori* (*H. pylori*), a gram-negative bacterium renowned for its pathogenic ability, is distinguished by the presence of polar flagella, facilitating its motility. Predominantly localized within the gastric epithelium, the bacterium cannot be easily and effectively eradicated (Vaziri et al., 2018; Sterbenc et al., 2019). Acute *H. pylori* infection is characterized by symptoms resembling gastritis, including upper abdominal pain, nausea, vomiting, and bloating (Zhang et al., 2022). The most common infection is a chronic process without obvious symptoms. Chronic *H. pylori* carriage is frequently asymptomatic but has significant implications for the development of many gastrointestinal diseases, including gastritis, gastric and duodenal ulcers, gastrointestinal mucosa-associated lymphoma, and gastrointestinal carcinoma (cancer) (Wang et al., 2014; Malfertheiner et al., 2023; Ralser et al., 2023). *H. pylori* is the most significant risk factor for gastrointestinal malignancy. Its presence increases the likelihood of developing gastric cancer (GC) by 2.7 to 12 times. Without *H. pylori* infection, between 35% and 89% of GC cases might not have occurred (Ralser et al., 2023). *H. pylori* infection can cause abnormal expression of carcinogenic genes, tumor suppressor genes, cell-cycle regulating genes, cell adhesion molecules, and other factors, leading to malignant progression of GC (Ishaq and Nunn, 2015). In addition, virulent toxins produced by *H. pylori* are key risk factors of malignant transformation. Toxic factors enter gastric mucosal epithelial cells to affect substance transport and signal transmission, prevent cell repair, and promote malignant proliferation (Diaz et al., 2018). Moreover, *H. pylori* infection can induce tumor related inflammatory responses, leading to malignant lesions (Yang et al., 2014a). *H. pylori* infection may also influence epigenetic regulations by altering the expression of noncoding RNAs including microRNAs(miRNA) and long noncoding RNAs (lncRNAs).

LncRNAs are RNAs with more than 200 nucleotides (Liu et al., 2022b). Despite their inability to encode proteins, they play intricate and diverse regulatory roles (Nojima and Proudfoot, 2022). LncRNAs can modulate a range of biological processes, including transcription, translation, cellular structural integrity, cell cycle, apoptosis, and stem cell pluripotency (Martianov et al., 2007; Rinn et al., 2007; Huarte et al., 2010; Herman et al., 2022; Zhou et al., 2022; Liu et al., 2022b). It has been established that lncRNAs play crucial roles in the processes of *H. pylori* infection and subsequently contribute to the pathogenesis of gastrointestinal cancers (Zhou et al., 2016; Liu et al., 2020; Wang et al., 2020; Rao et al., 2021). In this paper, we will elucidate the processes of *H. pylori* infection and its treatment methods, discuss the role of *H. pylori* in gastrointestinal cancer progression, provide an overview of lncRNA classification and functional mechanisms, and delve into the functions of lncRNAs in *H. pylori*-related gastrointestinal cancers.

## 2 *H. pylori* characterization

### 2.1 Transmission of *H. pylori*

The typical clinical progression of *H. pylori* infection follows this pattern (Wang et al., 2014; de Brito et al., 2019; Cho et al., 2021): *H. pylori* initially colonizes and infects the gastric mucosa following oral introduction, leading to chronic and superficial gastritis within a few weeks or month. Over the course of years to decades, this infection may progress to manifest conditions such as duodenal ulcers, gastric ulcers, and chronic atrophic gastritis.

**Figure 1:**
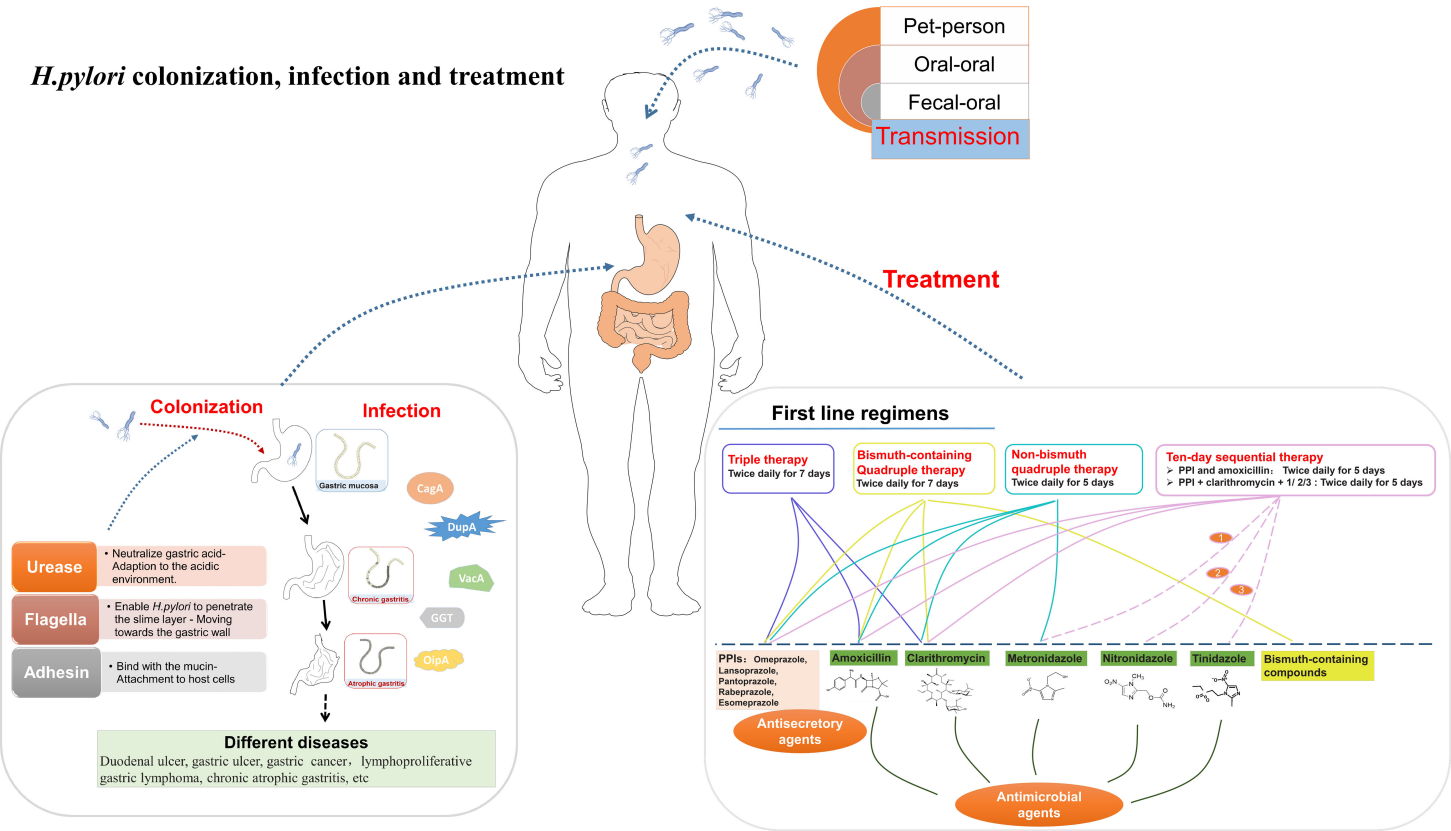
A summary diagram about the transmission, colonization, infection of *H. pylori*.

The most plausible routes of transmission include oral-oral, fecal-oral, and pet-to-person transmission (Duan et al., 2023) (**Figure 1**). Oral-oral transmission is the main way of *H. pylori* transmission, illuminating the observed familial aggregation of *H. pylori* infections (de Brito et al., 2019). Fecal-oral transmission is primarily associated with poor basic hygiene conditions and the consumption of contaminated water (Matsuzaki et al., 2021). Consequently, elevating economic levels and living conditions can diminish the incidence of *H. pylori* infection (Camilo et al., 2017).

### 2.2 Colonization and infection of *H. pylori*

There are countless proton pumps on the membrane of parietal cell, which render the stomach intensely acidic, characterized by a pH range of 0.9 to 1.5 (de Brito et al., 2019; Oztekin et al., 2021). Nonetheless, *H. pylori* exhibits a remarkable ability to penetrate this formidable acidic barrier and successfully colonize the stomach. The process of *H. pylori* colonization and infection can be divided into four stages (Camilo et al., 2017; Huang et al., 2017; Malfertheiner et al., 2023; Salvatori et al., 2023) (**Figure 1**): (1) Adaption to the acidic gastric environment; (2) Migration towards the gastric wall; (3) Attachment to host cells; (4) Toxin release and tissue damage.

*H. pylori* initiates its transmission by adapting to the hostile acidic environment of the stomach. In this process, *H. pylori* employs key enzymes, including hydrogenase and urease. Urease catalyzes the hydrolysis of urea to generate ammonia (NH_3_) and carbon dioxide (CO_2_) (Weeks et al., 2000; Camilo et al., 2017; Yuen et al., 2017). The alkaline NH3 then neutralizes the acidic gastric environment, effectively enabling *H. pylori* to endure this acidic environment and facilitating the infection processes (Camilo et al., 2017; Yuen et al., 2017; Salvatori et al., 2023). Hydrogenase is a component of a signaling pathway that allows *H. pylori* to utilize molecular hydrogen (H_2_) as an energy source for metabolic processes (Olson and Maier, 2002).

The bacterium then moves towards the gastric wall, where it will ultimately establish its niche. The movement of *H. pylori* is critically dependent on its flagellar structure. The remarkable motility conferred by these flagella allows the bacterium to efficiently traverse the protective mucous layer covering the gastric mucosa (Camilo et al., 2017; Loconte et al., 2017; Zhang et al., 2017). Mutations in the flagellar genes can render *H. pylori* incapable of infecting hosts (Camilo et al., 2017; Zhang et al., 2017). Notably, higher levels of acidity are associated with swifter flagellar movement (Camilo et al., 2017; Salvatori et al., 2023). This suggests that flagella can, to some extent, provide protection to the bacterium from the hostile acidic gastric environment.

After reaching the gastric wall, *H. pylori* proceeds to establish adhesion to host cells. *H. pylori* adhesin molecules bind with mucin and the receptors on the surface of gastric mucosa (Yamaoka et al., 2002; Acio-Pizzarello et al., 2017; Gur et al., 2019). These adhesive interactions firmly anchor the bacterium in place. This adhesion mechanism have dual implications: Firstly, it shields the bacteria from the clearance mechanisms and the renewal of the protective gastric mucous layer (Gur et al., 2019; Ndzouboukou et al., 2021); Secondly, it facilitates the material exchange between the bacteria and host cells, including nutrient absorption and toxin transportation (Yamaoka et al., 2002; Ndzouboukou et al., 2021).

Finally, *H. pylori* releases virulent toxins, causing a series of reactions that result in significant tissue damage (Abedrabbo et al., 2017). These virulent toxins include cytotoxic associated antigen A (CagA), duodenal ulcer promoting gene A protein (DupA), vacuolating cytotoxic (VacA), gamma-glutamyl transpeptidase (GGT), and outer inflammatory protein (OipA) (Yamaoka et al., 2000; Fischer, 2011; Boquet and Ricci, 2012; Alam et al., 2020; Meng et al., 2021). CagA modifies epithelial cell morphology and disrupts cell polarity (Hatakeyama, 2014). Additionally, CagA modulates cell adhesion and migration processes (Higashi et al., 2002). *H. pylori* strains exhibiting elevated CagA activity are correlated with an increased risk of gastric adenocarcinoma progression (Tsutsumi et al., 2003; Suzuki et al., 2009; Salvatori et al., 2023). DupA protein is an important contributor to the acid resistance of *H. pylori* and can upregulate interleukin-8 (IL-8) in the antral gastric mucosa. IL-8 induction initiates inflammation, resulting in gastritis and duodenal ulcers (Lu et al., 2005; Cho et al., 2021; Malfertheiner et al., 2023; Salvatori et al., 2023). Active DupA within *H. pylori* strains can facilitate the development of gastric carcinoma (Oztekin et al., 2021; Malfertheiner et al., 2023). VacA is a pivotal protein in *H. pylori*’s pathogenicity (de Brito et al., 2019; Duan et al., 2023). VacA destroys mitochondria function and affects many membranous structures, ultimately leading to the collapse of gastric epithelial cells (Yamasaki et al., 2006; Salvatori et al., 2023). Moreover, VacA also enhances immune tolerance and promotes *H. pylori*’s persistent infection (Terebiznik et al., 2006; Djekic and Muller, 2016). The cumulative effects of VacA exacerbates gastritis, as well as the development of ulcers and different cancers (Boquet and Ricci, 2012). GGT is a N-terminal nucleophile hydrolase produced by *H. pylori* (Shibayama et al., 2007). The enzymatic activity of GGT results in the generation of reactive oxygen species (ROS) which can disrupt the cell cycle, apoptosis and necrosis (Kim et al., 2010). GGT activity is found to be higher in peptic ulcer patients compared with those with other gastroduodenal diseases (Kim et al., 2010). OipA upregulates IL-8 levels, thus enhancing adhesion and inflammation (Farzi et al., 2018). The active form of OipA in *H. pylori* is associated with increased gastric pathogenicity (Sallas et al., 2019). In additional, PqqE, a *H. pylori* protease, is also considered a virulent factor (Marques et al., 2021). It can cleave junctional adhesion molecule A (JAM-A), a transmembrane protein implicated in regulating the epithelial cell barrier and cell polarity, to destroy gastric epithelial integrity (Marques et al., 2021). The virulent factors collectively contribute to the pathogenesis and pathological consequences associated with *H. pylori* infection.

### 2.3 *H. pylori* infection and gastric cancer development

Based on current knowledge on *H. pylori* infection, there are two main types of mechanisms by which *H. pylori* infection eventually cause gastrointestinal cancers. The indirect way refers to a chronic inflammatory response that can enhance cell turnover and abnormal mitosis. In the direct way, the bacterium can regulate the levels of many functional genes to alter the behavior of the epithelial cells, leading to cellular stemness and malignant transformation.

#### 2.3.1 Indirect regulation through inflammatory responses

**Figure 2:**
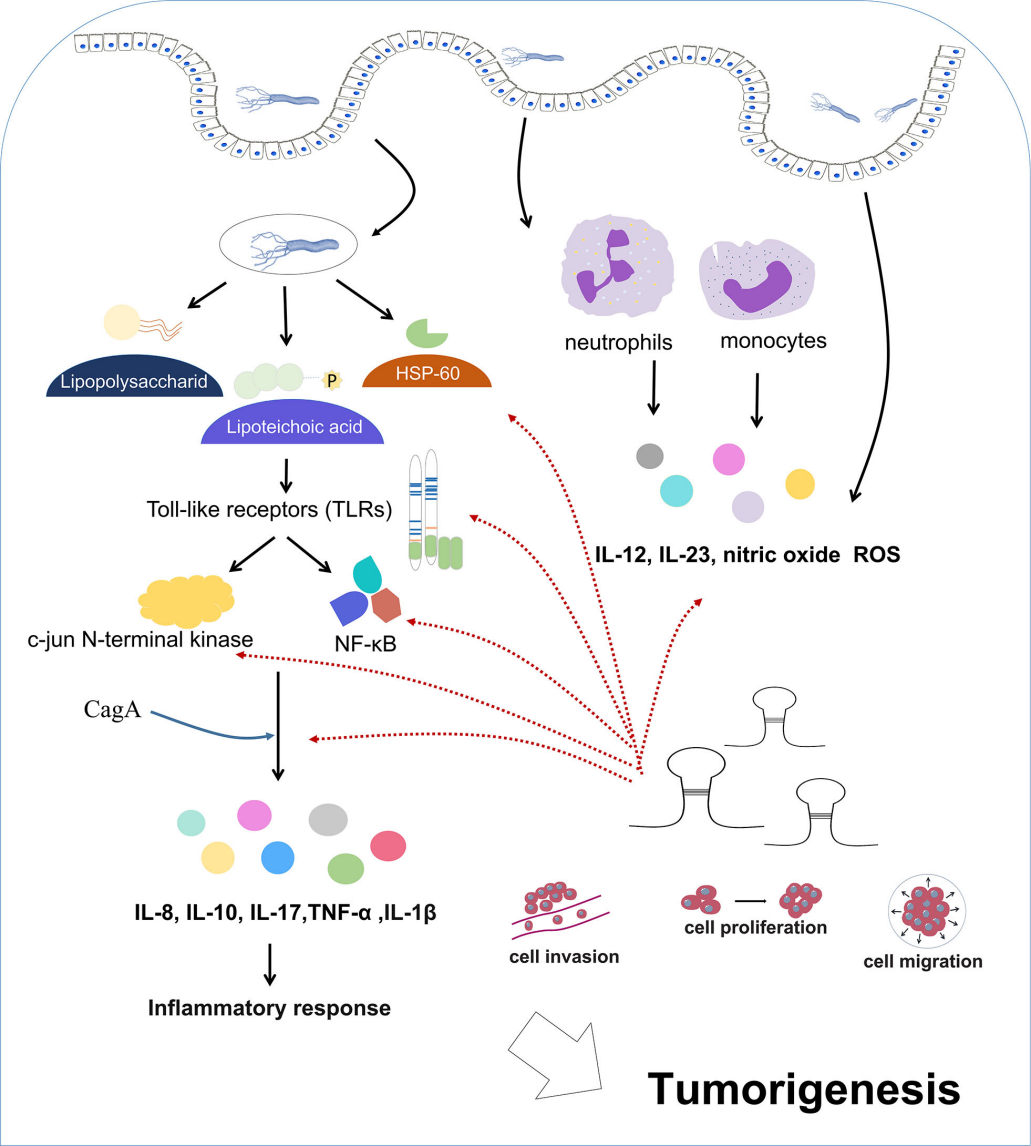
Immune reactions during *H. pylori* infection. *H. pylori* infection triggers a cascade of intricate immune reactions.

*H. pylori* infection triggers intricate immune reactions (Wang et al., 2014; Bagheri et al., 2018; Ralser et al., 2023) (**Figure 2**). Upon entering the gastrointestinal tract, *H. pylori-*carried antigens (e.g., lipopolysaccharide, lipoteichoic acid, HSP-60, etc) interact with the receptors on mucosal cells (toll-like receptors, TLRs) (Nagashima et al., 2015; Ram et al., 2015; Zhang et al., 2023). This interaction induces the activation of nuclear factor-kappa B (NF-κB) and c-jun N-terminal kinase (JNK) (Nagashima and Yamaoka, 2019), two promoter of the immune events that cause tumorigenesis. This activation enhances the release of proinflammatory cytokine, including IL-8, IL-10, IL-17, IL-1β and tumor necrosis factor α (TNF-α) (Devi et al., 2014; Merga et al., 2016; Nagashima and Yamaoka, 2019). These cytokines can mediate the activation, proliferation, and differentiation of specific T and B cells, thereby playing an important role in the inflammatory response and activation. Additionally, CagA addition facilitates the generation of cytokines dependent of NF-κB (Alandiyjany et al., 2017), thus intensifying proinflammatory responses that lead to tumorigenesis. Moreover, gastric mucosa is infiltrated by neutrophils and monocytes, resulting in the production of proinflammatory cytokine (IL-12, IL-23), nitric oxide and ROS (Mayadas et al., 2014; Kalisperati et al., 2017). *H. pylori* infection also induces the generation of immunoglobulins. In the serum of *H. pylori*-positive patients, specific IgM antibodies can be detected as early as 4 weeks after infection (Nurgalieva et al., 2005). Chronic infection also results in the production of immunoglobulins such as IgA and IgG. While chronic inflammation often presents asymptomatically, it elevates the risk of gastrointestinal diseases and predisposes individuals to gastric malignancy (Suerbaum and Michetti, 2002; Malfertheiner et al., 2023; Salvatori et al., 2023).

#### 2.3.2 Direct regulation through affecting gene functions

*H. pylori* infection directly influence many functional proteins in oncogenic pathways, such as virulent toxin, tumor suppressor protein 53 (TP53) (Fang et al., 2019), AT-rich-interaction domain 1A (ARID1A) (Fang et al., 2019), cyclooxygenase-2 (COX-2) (Martinson et al., 2020) and tyrosine kinase (Sonkar et al., 2020). Their dysregulation facilitate tumor promoting signaling, tumor initiation and progression. In the direct way, modulating virulent toxins such as CagA and VacA, plays a pivotal role. *H. pylori* infection increases CagA levels to cause multiple changes that damage cell function, eventually leading to cancer development (Rihane et al., 2021). *H. pylori* produces incisions in the extracellular claudin-8, E-cadherin, and occluded domains of epithelial cells through specific serine proteases, thereby enabling CagA to enter the gastric epithelial membrane (Marques et al., 2021). *H. pylori* strains with high CagA levels has been revealed to trigger the expression of activation induced deaminase (AID), a main regulatory enzyme for secondary antibody diversification, resulting in severe mutations in the genes encoding immunoglobulins (González et al., 2011). CagA can induce pro-inflammatory cytokine production by regulating various signaling pathways (Castaneda et al., 2019; Teresa et al., 2019). In addition, CagA can repress phosphatase activity and inactivate runt related transcription factor 3(RUNX3) that serves as a tumor inhibitor, leading to tumorigenesis (Sonkar et al., 2020). CagA has been shown to bind to integrin beta 1 (ITGB1), a receptor involved in anchoring *H. pylori* and host cell surface, leading to IL-8 generation by host cells (Zeng et al., 2020). VacA can disrupt epithelial barrier and inhibits T cell-mediated immune responses, prolonging the lifespan of *H. pylori* infection. Additionally, VacA can activate proinflammatory signaling pathways and act on the mitochondria to destroy the cell proliferation-apoptosis balance, causing cancer development (Azuma et al., 2004).

### 2.4 Methods of *H. pylori* diagnosis

Currently, there have been some methods to confirm *H. pylori* existence, including invasive methods and noninvasive methods (Fan et al., 2020). Rapid urease test (RUT)and Giemsa staining biopsy method are two widely used invasive methods in clinical. These two methods are based on gastroscopy examination in which a gastric mucosal specimen needs to be taken for subsequent testing. Urease testing in RUT and Giemsa staining will be performed to diagnose *H. pylori* infection. RUT is safe and accurate with a sensitivity of about 94%, specificity of about 95-100%, and an accuracy rate of 95% (Roy et al., 2016). Giemsa staining (direct smear) is easy to operate and is effective for rapid diagnosis. But it is prone to missed diagnosis with low bacterial abundance. The sensitivity and specificity of histopathological methods reach about 98% (Roy et al., 2016). Traditional Giemsa staining has been improved to solve problems such as high cost, long time-consumption, and complicated operation. The improved Giemsa staining method is simpler and time-saving with the same sensitivity as the traditional staining method. It has been proven to be superior to RUT detection (Fan et al., 2020). Therefore, this method can be used for routine clinical examinations to provide fast and accurate diagnosis. However, both methods belong to invasive examination and may cause discomfort during the process, so it cannot be used for all patients.

Urea breath test (UBT) with ^13^C or ^14^C, serum antibody detection, fecal antigen detection and nucleic acid detection based on real-time fluorescence quantitative polymerase chain reaction (RT-PCR) are the most commonly used noninvasive methods. UBT only requires subjects to takes ^13^C or ^14^C isotope labeled urea from breath. And then carbon atom in the CO_2_ exhaled by the subject’s breath will be detected (Skrebinska et al., 2018). The urease secreted by *H. pylori* will decompose urea into ammonia and CO_2_. The presence of ^13^C or ^14^C from exhaled CO_2_ indicates *H. pylori* infection. This method is highly accurate with sensitivity and specificity of around 95% (Skrebinska et al., 2018). In addition, it is convenient, fast, painless, and can avoid cross infection. Therefore, UBT is now considered the “gold standard” for diagnosing *H. pylori* infection. It has been extensively applied in clinical practice to determine the infection status of patients and follow-up after treatment. Serological antibody testing and fecal antigen detection method are dependent on antigen-antibody interaction (Skrebinska et al., 2018). They are simple, feasible, and inexpensive. However, serological antibody testing cannot distinguish whether patients have a previous infection or are currently infected (Park et al., 2002). Consequently, it is commonly used in epidemiological investigations of population infection. Fecal antigen detection results are usually based on past infections (Fan et al., 2020). In recent years, methods based on PCR, including multiplex PCR and RT-PCR, have also been widely used for the detection of *H. pylori* (Marrero Rolon et al., 2021).

### 2.5 Treatment of *H. pylori* infection

Currently, the standard approach for treating *H. pylori* infection usually involves a combined therapy consisting of antimicrobial and antisecretory agents (Yang et al., 2014b; de Brito et al., 2019) (**Figure 1**). Antisecretory agents can raise the PH level in the stomach (Yang et al., 2014b). The mainly used antisecretory agents are proton pump inhibitors (PPIs) (Sachs et al., 1995; Besancon et al., 1997; Yang et al., 2014b; Safavi et al., 2016). The primary antimicrobial agents used are antibiotics such as amoxicillin, clarithromycin, nitronidazole, metronidazole (Yang et al., 2014b; Safavi et al., 2016). Bismuth-containing compounds are also used in the treatment of *H. pylori* infection (Malfertheiner et al., 1997). Various agent combinations lead to different treatment outcomes (Yang et al., 2014b; Safavi et al., 2016). These treatment regimens include triple therapy, bismuth-containing quadruple therapy, non-bismuth quadruple therapy (concomitant therapy), sequential therapy and so on (Yang et al., 2014b; Safavi et al., 2016).

In the triple therapy, a standard dose of PPI, along with clarithromycin (500 mg) and amoxicillin (1 g), is administered twice daily for 7 days (World Gastroenterology, 2011; Malfertheiner et al., 2012). Bismuth-containing Quadruple therapy augments the triple therapy by introducing bismuth (120 mg) to the regimen (Malfertheiner et al., 1997). These two regimens are the first-line approaches for *H. pylori* eradication. To deal with the rising resistance to clarithromycin, non-bismuth quadruple therapy, involving the combination of metronidazole with elements of triple therapy (twice daily for 5 days), has been developed (Hu et al., 2017; Wani et al., 2018). This therapy elevates the treatment efficacy, achieving an eradication rate exceeding 90% (Megraud et al., 2013). Sequential therapy is also used in certain cases. In a ten-day sequential therapy (Zullo et al., 2000, Zullo et al, 2003), PPI and amoxicillin are administered for the first 5 day, followed by a 5-day triple therapy (PPI + clarithromycin + nitronidazole/metronidazole/tinidazole) (Hsu et al., 2011). This regimen can achieve an impressive eradication rate, peaking at 98%.

## 3 LncRNA classification and action mechanisms

**Figure 3:**
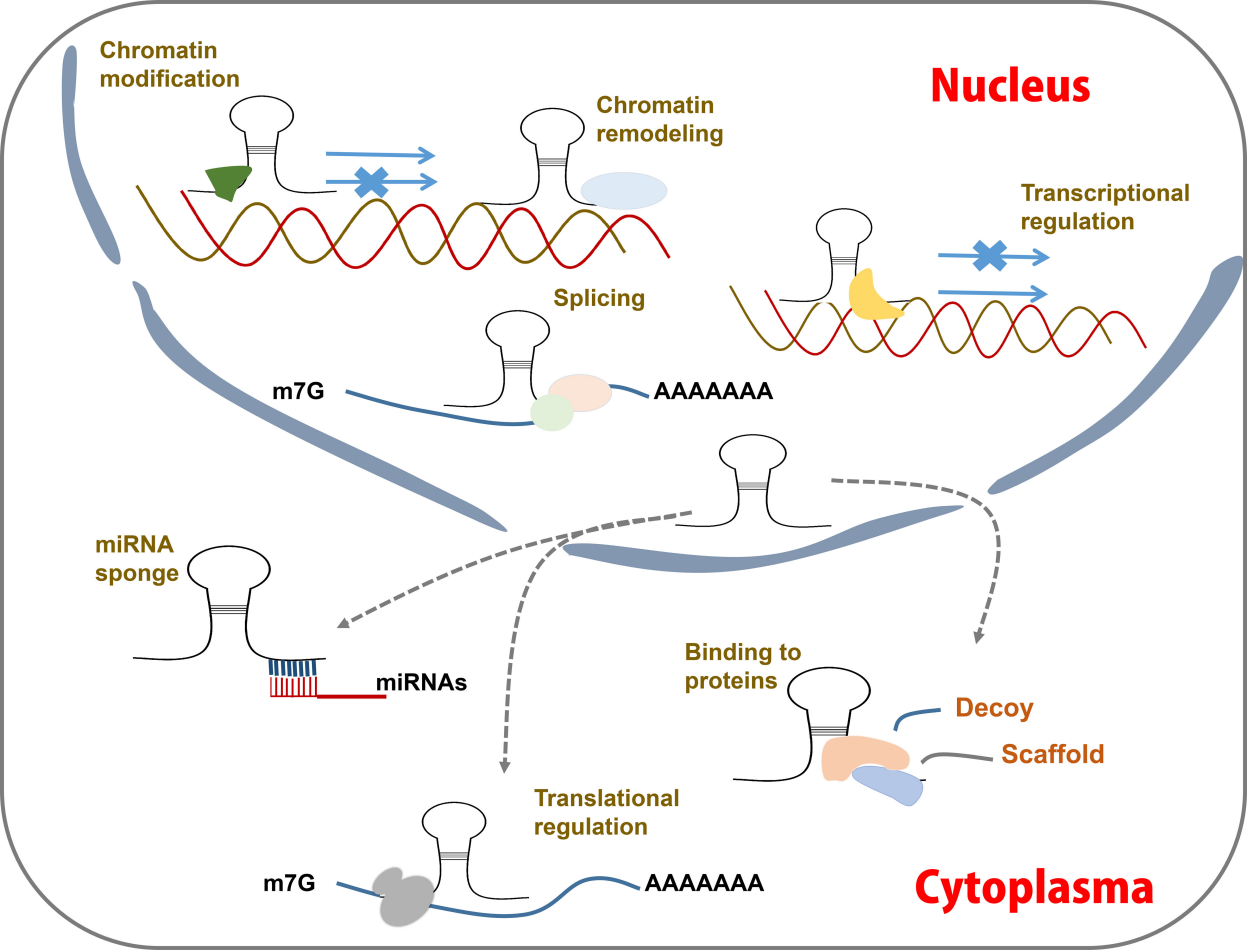
LncRNA action mechanisms. LncRNAs function by acting as guides, enhancers, scaffolds and decoy to regulating post-transcription. They can interact with proteins and miRNAs, modulate mRNA transcription, modify chromatin status, etc.

LncRNAs can be categorized into six primary types based on their initiation loci in relation to the protein-coding genes (PCGs) (**Figure 3**) (Sigova et al., 2013; Yang et al., 2019): sense lncRNAs, antisense lncRNAs, intronic lncRNAs, bidirectional lncRNAs, long intergenic noncoding RNAs (lincRNA), and enhancer RNAs. Among these, upstream antisense RNAs (60~70%) and enhancer RNAs (~20%) constitute the majority (Jarroux et al., 2017; Zhou et al., 2022; Liu et al., 2023). The transcription of antisense lncRNAs is initiated inside PCGs or from the 3’ UTR of PCGs (Wilusz et al., 2009). Intronic lncRNAs initiate their transcription within the introns of PCG and extend to cover these intronic regions (Wilusz et al., 2009). The transcription of bidirectional lncRNAs is initiated from the PCGs’ promoters. Intergenic lncRNAs are transcribed from regions between genes (Wilusz et al., 2009). Enhancer lncRNAs are generated from enhancer regions and play a role in the activation of specific enhancers (Yang et al., 2019; Bridges et al., 2021).

LncRNAs can directly bind to DNAs, RNAs, and proteins (Postepska-Igielska et al., 2015; Jiang et al., 2017; Zealy et al., 2018; Ao et al., 2023). Based on these interactions, lncRNAs can be classified into four distinct functional categories: guides, enhancers, scaffolds and decoys (Yang et al., 2019; Rao et al., 2021; Liu et al., 2022b) (**Figure 3**). Many lncRNAs function as guides, directing chromatin-modifying complexes such as the polycomb repressor complex 2 to specific genomic targets (Wilusz et al., 2009; Kotake et al., 2011; Yang et al., 2019), thereby mediating transcriptional inhibition. Some lncRNAs serve as enhancers by activating the expression of target genes (Redfern et al., 2013; Trimarchi et al., 2014). LncRNAs with scaffold functionality can act as molecular hubs, bringing together RNA-binding proteins in close spatial proximity or facilitating interactions with DNAs (Puvvula et al., 2014). Additionally, some lncRNAs function as decoys to sequester regulatory factors in either the cytoplasm or nucleus (Tripathi et al., 2010; Cesana et al., 2011).

## 4 LncRNAs in *H. pylori*-related gastrointestinal cancers

**Figure 4:**
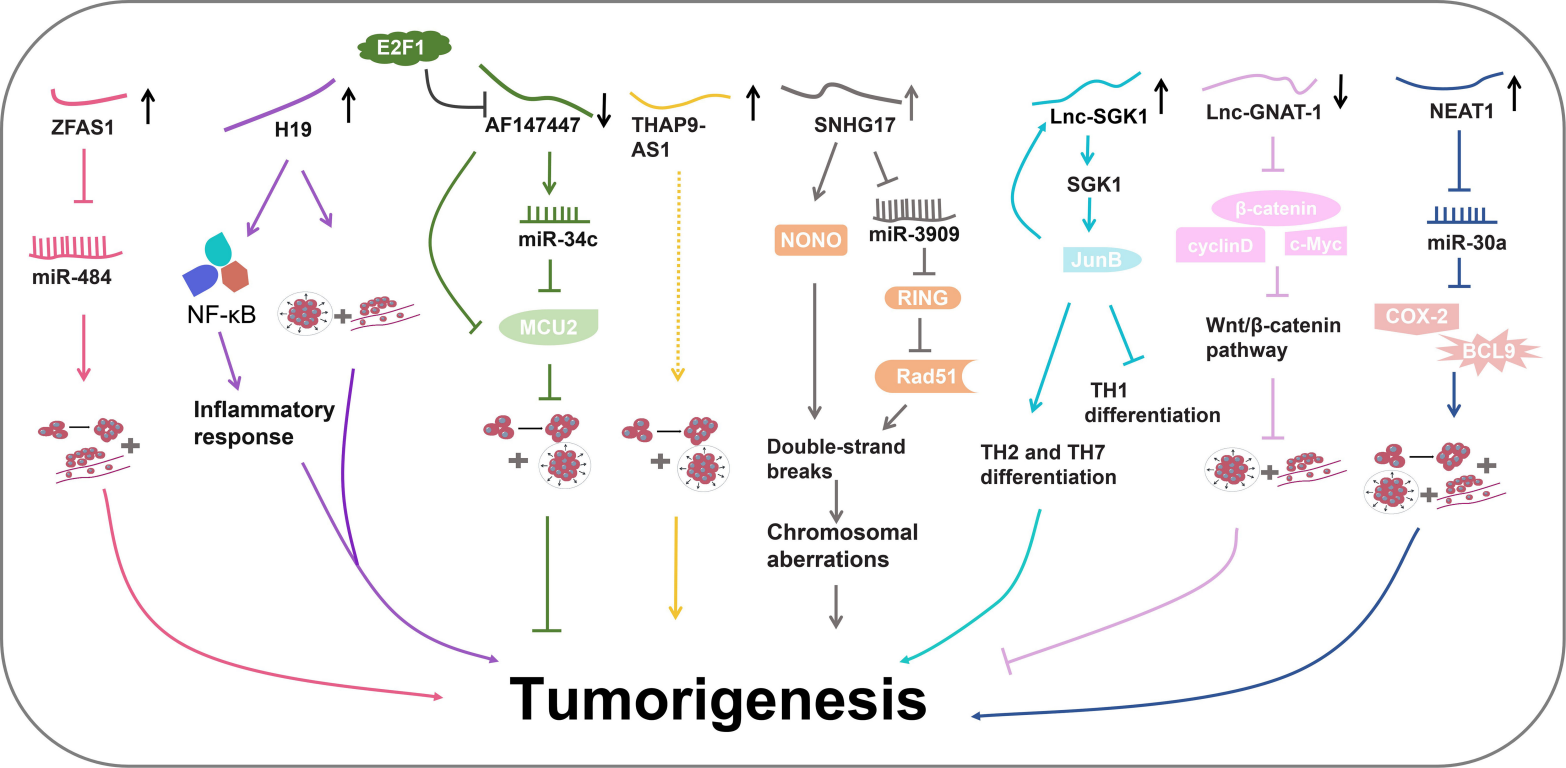
The regulatory pathways of lncRNAs in *H. pylori*-induced gastrointestinal cancer.

*H. pylori* is classified as a class I carcinogen in gastrointestinal cancers (Ralser et al., 2023; Salvatori et al., 2023). *H. pylori* infection can exacerbate chronic inflammation of the gastric mucosa and promote the deterioration of normal gastric mucosa into carcinoma (Dastmalchi et al., 2019; Mostaghimi et al., 2024). Recent studies have unveiled the involvement of lncRNAs in *H. pylori*-induced gastrointestinal cancers (**Figure 4**) (**Table 1**).

**Table 1:** LncRNAs in *H. pylori* -induced gastrointestinal cancers.

LncRNAs	Expression	Molecular mechanism	Effect	Reference
ZFAS1	Up	Target miR-484	Enhance	(Xie et al., 2018)
H19	Up	Promote NF-κB pathway and induce inflammation	Enhance	(Zhang et al., 2019)
AF147447	Down	Upregulate miR-34c and decrease MUC2 activity	Suppress	(Zhou et al., 2016)
THAP9-AS1	Up	Unknown	Enhance	(Jia et al., 2019)
SNHG17	Up	Disrupt the normal double-strand break (DSB) repair system, by interacting with NONO protein or by regulating miR-3909/RING1/Rad51 axis	Enhance	(Han et al., 2020).
Lnc-SGK1	Up	Enhance the differentiation of Th2 and Th17 cells via the SGK1/JunB axis.	Enhance	(Yao et al., 2016)
Lnc-GNAT1-1	Down	Disrupting Wnt/β- catenin pathway	Suppress	(Liu et al., 2018)
NEAT1	Up	Target the miR-30a-COX-2/BCL9 axis	Enhance	(Rao et al., 2021)
XLOC_004122 and XLOC_018438	Down	Unknown	Unknown	(Yang et al., 2015)
several lncRNAs	up	Unknown	Unknown	(Li et al., 2020a)
several lncRNAs	—	Unknown	Unknown	(Zhu et al., 2015)

### 4.1 ZFAS1

The LncRNA known as zinc finger antisense 1 (ZFAS1) is an antisense RNA transcribed from the 5’ end of the *Znfx1* gene (Xie et al., 2018). ZFAS1 has been revealed to participate in various diseases, including acute myocardial infarction and several types of cancer (Zhang et al., 2016; Gao et al., 2017; Xia et al., 2017). In a study by Xie et al., ZFAS1 was found to be highly expressed in both colorectal cancer (CRC) tissues and cell lines (Xie et al., 2018). ZFAS1 downregulation inhibited proliferation and invasion of CRC cells. Further experiments revealed its potential role as an oncogene by targeting miR-484. MiR-484 plays extensive roles in diverse cancers through interacting with various functional proteins including CRC and GC (Li et al., 2020b; Shen et al., 2021). Therefore, ZFAS1 might aggravate CRC by influencing miR-484 activity and its downstream pathways. Additionally, high ZFAS1 levels were revealed to be associated with severe *H. pylori* infection in CRC patients, indicating the diagnostic potential of ZFAS1 in *H. pylori* infection (Xie et al., 2018).

### 4.2 H19

LncRNA H19 has been identified as a regulatory molecule in tumorigenesis (Bartolomei et al., 1991; Hao et al., 2017). Zhang et al. have uncovered the role of H19 in promoting GC progression (Yan et al., 2017). They detected elevated levels of H19 in the serum of GC patients with *H. pylori* infection (Yang et al., 2016). Zhang and colleagues further elucidated the involvement of H19 in *H. pylori-*associated GC development (Zhang et al., 2019). *H. pylori* infection could enhance GC cell migration and invasion, concurrently elevating the levels of pro-inflammatory cytokine, thereby promoting GC progression and inflammatory responses. H19 further amplified the impact of *H. pylori* infection in GC development (Zhang et al., 2019). Subsequent experiments revealed that H19 might induce inflammation by the NF-κB pathway, which is the most important inflammatory signaling pathway in cancer. It triggers the production of various inflammatory factors by cancer cells and inflammatory cells, thereby igniting inflammation and promoting cancer cell proliferation, survival, deterioration, and metastasis (Zinatizadeh et al., 2021). Consequently, H19 could enhance GC cell growth, migration and invasion induced by *H. pylori* infection via promoting NF-κB-induced inflammation (Zhang et al., 2019).

### 4.3 AF147447

Zhou et al. conducted a microarray using *H. pylori*-positive and negative gastric tissues (Zhou et al., 2016). They discovered that lncRNA-AF147447 was downregulated in *H. pylori*-infected gastric tissues. Lower levels of AF147447 were associated with a more severe pathological condition (Zhou et al., 2016). AF147447 overexpression suppressed gastric cell proliferation and migration, indicating its anti-tumor role in *H. pylori-*induced GC progression. Further investigations unveiled that AF147447 could directly bind to both mucin 2 (MUC2) protein and miR-34c (Zhou et al., 2016). MUC2 is a glycosylated protein that is specifically expressed in the gastrointestinal tract (Walsh et al., 2013). MUC2 has been found to be related to the progression of CRC and ovarian cancer (He et al., 2013; Walsh et al., 2013). The high expression of MUC2 indicates poor prognosis (He et al., 2013; Kim et al., 2013). Importantly, miR-34c was found to directly target MUC2 protein. MUC2 levels were significantly increased in *H. pylori-*infected gastric tissues. AF147447 overexpression decreased MCU2 levels and increased miR-34c expression (Zhou et al., 2016). Detailed mechanistic exploration demonstrated that AF147447 could directly attenuate MCU2 activity or by upregulating miR-34c. Furthermore, it was observed that the reduced levels of AF147447 caused by *H. pylori* infection were regulated by the transcription factor E2F1 that plays an important role in controlling cell cycle and anti-tumor gene function (Zhou et al., 2016). Collectively, these findings suggest that AF147447 can suppress gastric tumorigenesis by decreasing MUC2 activity via the upregulation of miR-34c (Zhou et al., 2016).

### 4.4 THAP9-AS1

Jia et al. performed an RNA-seq using *H. pylori-*infected GC cells and controls cells (Jia et al., 2019). They discovered a significant upregulation of lncRNA-THAP9-AS1 in *H. pylori-*infected GC cells. THAP9-AS1 was also found to exhibit higher expression levels in *H. pylori*-positive GC tissues (Jia et al., 2019). Further experiments demonstrated that THAP9-AS1 could enhance the proliferation and migration of GC cells (Jia et al., 2019). *H. pylori* infection induced the expression of THAP9-AS1 and enhanced GC cell proliferation and migration, whereas THAP9-AS1 knockdown reversed these effects (Jia et al., 2019). Bioinformatics prediction discovered two possible targets of THAP9-AS1, SEC31A and THAP9. However, the specific underlying mechanism has not been explored and more studies are required. All findings demonstrate that *H. pylori* might promote GC tumorigenesis by upregulating THAP9-AS1 levels (Jia et al., 2019).

### 4.5 SNHG17

A microassay was carried out to examine the lncRNA profiles in *H*. *pylori-*negative normal gastric epithelial tissues, *H*. *pylori-*positive gastritis tissues, and *H. pylori-*positive GC tissues (Han et al., 2020). It was discovered that lncRNA small nucleolar RNA host gene 17 (SNHG17) was highly expressed in *H*. *pylori-*positive GC tissues. SNHG17 was also upregulated in the *H. pylori-*infected gastric epithelial cells. Elevated levels of SNHG17 might indicate poorer overall survival rate (Han et al., 2020). More experiments revealed that *H*. *pylori* could induce SNHG17 expressions. SNHG17 was found to disrupt the normal double-strand break (DSB) repair system, leading to an increase in DSB formation and subsequent chromosomal aberrations (Han et al., 2020). Mechanism exploration showed that SNHG17 facilitated the repair of DSBs through nonhomologous end-joining (NHEJ) by interacting with non-POU domain-containing octamer-binding protein (NONO), a RNA- and DNA-binding nuclear factor involved in DNA damage repair. Moreover, SNHG17 might also bind to miR-3909 to modulate the activity of the ring finger protein 1 (RING1)/Rad51 axis (Han et al., 2020), thereby converting the DSB repair balance from homologous recombination (HR) to NHEJ, causing genomic disorders. Taken together, *H*. *pylori* infection might promote GC tumorigenesis by upregulating SNHG17, which in turn impairs genome stability by interacting with NONO protein or by regulating miR-3909/RING1/Rad51 axis (Han et al., 2020).

### 4.6 Lnc-SGK1

The *serum and glucocorticoid-inducible kinase 1* (*SGK1*) gene plays a pivotal role in the development of several cancers (Lang and Shumilina, 2013; Lang and Stournaras, 2013). SGK1 protein has been found to participate in the differentiation of T cells, such as T helper (TH) cell 2 and 17 (TH2, TH17) (Wu et al., 2013; Heikamp et al., 2014). In a study by Yao et al., a novel lncRNA produced from the *SGK1* gene, referred to as Lnc-SGK1, was identified (Yao et al., 2016). Lnc-SGK1 was shown to function as a *cis*-regulatory factor capable of promoting SGK1 expression. In peripheral T cells of *H. pylori*-positive patients, both SGK1 and Lnc-SGK1 exhibited increased levels compared with *H. pylori*-negative patients (Yao et al., 2016). Moreover, *in vitro* experiments demonstrated that SGK1 was upregulated in T cells upon *H. pylori* infection. The elevated expression of SGK1 activated Jun B proto-oncogene (JunB), a transcriptional factor, leading to an increase in Lnc-SGK1 levels. Lnc-SGK1, in this *cis-*regulatory feedback loop, further promoted SGK1 expression (Yao et al., 2016). Lnc-SGK1 was revealed to facilitate TH2 and TH7 differentiation and suppress TH1 differentiation by regulating the SGK1/JunB signaling pathway. Elevated levels of *H. pylori* infection-related Lnc-SGK1 might indicate a poor prognosis for GC (Yao et al., 2016). Altogether, these observations show that Lnc-SGK1 can be induced by *H. pylori* infection and enhance the differentiation of Th2 and Th17 cells in GC via the SGK1/JunB axis.

### 4.7 Lnc-GNAT1-1

LncRNA-GNAT1 (G protein subunit α transducin 1)-1 has previously been reported to exert a suppressive effect on colorectal cancer (Ye et al., 2016). Liu et al. investigated its role in *H. pylori* infection-induced GC. In GC cell lines, *H. pylori* infection significantly reduced the expression of lnc-GNAT-1 (Liu et al., 2018). Overexpression of Lnc-GNAT-1 in *H. pylori*-infected GC cells could repress cell migration and invasion. Furthermore, Lnc-GNAT-1 overexpression also suppressed GC tumor growth *in vivo* (Liu et al., 2018). Lnc-GNAT-1 overexpression notably decreased protein expression in Wnt/β-catenin pathway which mediates tumor cell stemness and thereby promote tumorigenesis (Liu et al., 2022a). In conclusion, *H. pylori* infection can decrease Lnc-GNAT-1 levels and lnc-GNAT-1 might inhibit GC progression by disrupting Wnt/β-catenin pathway (Liu et al., 2018).

### 4.8 NEAT1

LncRNA nuclear para-speckle assembly transcript 1 (NEAT1) has been reported to regulate various diseases (Rao et al., 2021). Rao et al. found that NEAT1 was associated with *H. pylori* infection-induced GC. NEAT1 levels were increased in *H. pylori*-infected GC tissues (Rao et al., 2021). NEAT1 overexpression promoted the proliferation, invasion and migration of *H*. *pylori*-infected GC cell lines (Rao et al., 2021). Furthermore, NEAT1 was found to sponge miR-30a (miR-30a-3p and miR-30a-5p) to suppress its expression. MiR-30a can interact withCOX-2 and B-cell lymphoma 9 (BCL9), inhibiting their expression (Rao et al., 2021). COX-2 is a promoter of many cancers and can be a biomarker for cancer prognosis (Hashemi Goradel et al., 2019). BCL9 also can promote tumor progression (Wu et al., 2024). NEAT1 increased the expression of COX-2 and BCL9. The levels of COX-2 and BCL9 were also elevated in *H. pylo*ri-infected GC tissues. All in all, these findings suggest that NEAT1 may promote *H. pylori* infection-induced GC by the miR-30a-COX-2/BCL9 axis (Rao et al., 2021).

### 4.9 Integrative analysis of *H. pylori* infection diseases

Despite the discovered functional lncRNAs related with *H. pylori* infection, there are still many lncRNAs obtained through integrative analysis with unknown action mechanisms. Liu et al. constructed several cross-networks involving lncRNAs, mRNAs, miRNAs and proteins in *H. pylori* infection-induced diseases, such as atrophic gastritis (GA) and GC (Liu et al., 2020). In another study, a whole-transcriptome sequencing was conducted in *H. pylori-*infected GC cells and several upregulated lncRNAs and mRNAs were identified (Li et al., 2020a). Zhu and colleagues performed a microarray analysis in *H. pylori-*infected gastric epithelial cells (Zhu et al., 2015) and found some aberrantly expressed lncRNAs. Informatics analyses revealed these lncRNAs might lead to pathogenesis of *H. pylori*-induced disorders and diseases (Zhu et al., 2015). Yang et al. also found many lncRNAs with altered expression in *H. pylori-*infected gastric epithelial cells using microarray analysis (Yang et al., 2015). Among these lncRNAs, XLOC_004122 and XLOC_018438 levels were reduced in *H. pylori*-positive patients (Yang et al., 2015). Further findings suggested that XLOC_004122 and XLOC_018438 might be related to the immune responses of host to *H. pylori* infection (Yang et al., 2015).

## 5 LncRNAs as predictors of *H. pylo*ri infection

LncRNAs have been shown to have diagnostic potential for *H. pylori* infection. lncRNA NR_026827 was found to be significantly downregulated in gastric epithelial cells infected with *H. pylori.* In addition, NR_026827 showed reduced expression in all stages of GC related to *H. pylori* infection (Zhong et al., 2018), suggesting its value as a diagnostic marker for *H. pylori* infection (Zhong et al., 2018). Through integrated informatics analysis, Yang et al. identified two significantly upregulated lncRNAs, RP11-169F17.1 and RP11-669N7.2, in stomach adenocarcinoma (Yang and Song, 2019). Elevated levels of these lncRNAs were associated with poor overall survival, suggesting their prognostic potential (Yang and Song, 2019). They were also found to have an intimate relationship with *H. pylori* infection-induced gastrointestinal diseases, such as gastritis, duodenal ulcer and GC (Yang and Song, 2019). Xin et al. explored a series of differentially expressed lncRNAs in *H. pylori*-infected GC tissues (Xin et al., 2021). Using informatics tools, they constructed lncRNA regulatory networks and identified 13 dynamically expressed lncRNAs that might be prognostic predictors for *H. pylori*-infected GC (Xin et al., 2021). Yang et al. reported increased levels of 2 lncRNAs, H19 and LINC00152, in GC patients. They found that higher expression of these 2 lncRNAs might suggest higher GC risks (Yang et al., 2016). Moreover, they discovered that the subjects with both *H. pylori* infection and high H19/LINC00152 levels might have elevated GC risks (Yang et al., 2016). Therefore, H19/LINC00152 may be potential prognostic indicators for GC patients with *H. pylori* infection (Yang et al., 2016). LncRNA MEG3 and HOTAIR were revealed to have significantly higher expression levels in *H. pylori-*negative GC patients than that in *H. pylori-*positive GC patients (Amini et al., 2022), indicating a negative correlation with *H. pylori* infection. Thereby, they might be diagnostic predictors for *H. pylori*-associated GC.

## 6 The clinical perspective and limitation of lncRNAs in *H. pylori* infection

Currently, there have been significant advancements in understanding and treating *H. pylori* infection. However, the incidence of gastrointestinal diseases caused by *H. pylori* infection has not decreased. Therefore, the persistent challenge still lies in the lack of effective and rapid diagnostic approach. Current detection methods for *H. pylori* infection predominantly require hospital-based procedures, consuming both time and financial resources. Consequently, there is an urgent need to develop more efficient and accessible diagnostic strategies. Among the currently used methods, fast diagnostic techniques like the test strip method and biological probes offer feasible applications. The test strip method is known for its simplicity and efficiency. For instance, in early pregnancy and blood glucose testing, only a few drops of urine or blood are needed and the results are displayed within seconds. Its user-friendly operation allows for easy self-administration, enabling quick initial diagnoses at home. Moreover, during the COVID-19 pandemic, the use of household test strip method significantly enhanced the diagnosis of COVID-19.

Given the complexity of *H. pylori* infection, there is a need to identify more efficient biomarkers for its rapid and convenient diagnosis. Studies have indicated the potential of lncRNAs as predictive markers for *H. pylori*-related gastrointestinal cancer. Due to the complicated secondary structures, protection of exosomes during transportation, and various post-transcriptional modifications, plasma or serum lncRNAs are more tolerant to degradation caused by repeated freeze-thaw cycles and room temperature. LncRNAs are widely present in mammals, and function in cell proliferation, differentiation, and individual growth and development. Compared with other types of biomarkers such as circulating tumor cells (CTCs), cfDNA, and exosomes, the stability and high expression level of circulating lncRNAs make it more advantageous as a potential biomarker. However, current methods for detecting lncRNAs, primarily relying on RT-PCR, are time-consuming (often taking more than an hour) and demand specialized equipment typically available only in hospital or lab settings. Therefore, developing test strips based on lncRNA sequences might be a more practical method. Alternatively, the use of stable and functional biological probes, such as golden nanocomposite probes and bioluminescence probes, might also be a feasible method. It might be a promising diagnostic choice to produce nucleic acid probes or test strips followed by the development of rapid detection kits using these probes and test strips.

However, despite the current findings, there are still several noteworthy gaps and problems that should be solved. Firstly, more comprehensive and systematic studies are needed to elucidate the complicated regulatory networks involving lncRNAs and *H. pylori*. Secondly, further investigation is required to validate the potential of lncRNAs as diagnostic or therapeutic targets. Thirdly, the association between lncRNAs and various virulence factors of *H. pylori* such as cagA and VacA also needs to be clarified in various gastrointestinal diseases.

In conclusion, lncRNAs are involved in the pathogenesis of *H. pylori*-associated gastrointestinal cancers via various pathways. They might be possible predictive markers and therapeutic targets. Nonetheless, further investigations are required to confirm and advance the clinical applicability of lncRNAs.

## Author contributions

LZ: Conceptualization, Data curation, Funding acquisition, Resources, Validation, Writing – original draft, Writing – review & editing. FY: Data curation, Validation, Writing – review & editing. YZ: Formal analysis, Validation, Writing – original draft. PL: Supervision, Validation, Writing – review & editing.
